# Chloroplast His-to-Asp signal transduction: a potential mechanism for plastid gene regulation in *Heterosigma akashiwo *(Raphidophyceae)

**DOI:** 10.1186/1471-2148-7-70

**Published:** 2007-05-03

**Authors:** Melinda R Duplessis, Kenneth G Karol, Elinor T Adman, Lauren YS Choi, Michael A Jacobs, Rose Ann Cattolico

**Affiliations:** 1 Department of Biology, University of Washington, Box 355325, Seattle, WA 98195-5325, USA; 2 Department of Biological Structure, University of Washington, Box 357420, Seattle, WA 98195-7420, USA; 3 Department of Medicine, University of Washington Genome Center, University of Washington, Box 352145, Seattle, WA 98195-2145, USA

## Abstract

**Background:**

Maintenance of homeostasis requires that an organism perceive selected physical and chemical signals within an informationally dense environment. Functionally, an organism uses a variety of signal transduction arrays to amplify and convert these perceived signals into appropriate gene transcriptional responses. These changes in gene expression serve to modify selective metabolic processes and thus optimize reproductive success. Here we analyze a chloroplast-encoded His-to-Asp signal transduction circuit in the stramenopile *Heterosigma akashiwo *(Hada) Hada *ex *Y. Hara *et *Chihara [syn. *H. carterae *(Hulburt) F.J.R. Taylor]. The presence, structure and putative function of this protein pair are discussed in the context of their evolutionary homologues.

**Results:**

Bioinformatic analysis of the *Heterosigma akashiwo *chloroplast genome sequence revealed the presence of a single two-component His-to-Asp (designated Tsg1/Trg1) pair in this stramenopile (golden-brown alga). These data represent the first documentation of a His-to-Asp array in stramenopiles and counter previous reports suggesting that such regulatory proteins are lacking in this taxonomic cluster. Comparison of the 43 kDa *H. akashiwo *Tsg1 with bacterial sensor kinases showed that the algal protein exhibits a moderately maintained PAS motif in the sensor kinase domain as well as highly conserved H, N, G_1 _and F motifs within the histidine kinase ATP binding site. Molecular modelling of the 27 kDa *H. akashiwo *Trg1 regulator protein was consistent with a winged helix-turn-helix identity – a class of proteins that is known to impact gene expression at the level of transcription. The occurrence of Trg1 protein in actively growing *H. akashiwo *cells was verified by Western analysis. The presence of a PhoB-like RNA polymerase loop in Trg1 and its homologues in the red-algal lineage support the hypothesis that Trg1 and its homologues interact with a sigma 70 (σ^70^) subunit (encoded by *rpoD*) of a eubacterial type polymerase. Sequence analysis of *H. akashiwo rpoD *showed this nuclear-encoded gene has a well-defined 4.2 domain, a region that augments RNA polymerase interaction with transcriptional regulatory proteins and also serves in -35 promoter recognition. The presence/loss of the His-to-Asp pairs in primary and secondary chloroplast lineages is assessed.

**Conclusion:**

His-to-Asp signal transduction components are found in most rhodophytic chloroplasts, as well as in their putative cyanobacterial progenitors. The evolutionary conservation of these proteins argues that they are important for the maintenance of chloroplast homeostasis. Our data suggest that chloroplast gene transcription may be impacted by the interaction of the His-to-Asp regulator protein (which is less frequently lost than the sensor protein) with the RNA polymerase σ^70 ^subunit.

## Background

One of the simplest mechanisms for controlling gene transcription response to environmental cues is mediated through a "two component" or "His-to-Asp" signal transduction system. In its most minimal configuration, this system is composed of two polypeptides, a sensor kinase and a response regulator protein that communicate via phosphotransfer events [reviewed in [1,2]]. A phosphoryl group is moved from a conserved histidine residue within the sensor kinase protein to an aspartic acid residue on its cognate response regulator. The response regulator impacts transcriptional activity by influencing promotor selection via its interaction with selected DNA targets [3] and with RNA polymerase [4,5]. His-to-Asp signal transduction systems were first characterized in *Escherichia coli *[6-8]. In eubacteria, 0 to more than 300 His-to-Asp proteins have been shown to occur [see [9-11] for discussion]. Recent complete genome sequencing endeavours document that the cyanobacterial genomes of *Synechocystis *sp. PCC 6803 and *Anabaena *sp. PCC 7120 contain about 80 and 208 general signal transduction proteins, respectively [12,13].

Given the endosymbiotic origin of plastids from a cyanobacterial-like ancestor [14,15], it is not surprising that genes for His-to-Asp signal transducers have been found encoded in chloroplasts. In this study, the wall-less, unicellular alga *Heterosigma akashiwo *is used as a model system for analyzing a chloroplast-encoded, two-component, signal transduction system. Each *H. akashiwo *cell contains approximately 30 discoidal chloroplasts [16]. These organelles are surrounded by four membranes, indicative of their serial endosymbiotic origin from a putative rhodophytic ancestor [14,17]. As an obligate autotroph, *H. akashiwo *is dependent on chloroplast function for survival and responds to changing environmental cues by rapidly altering transcript levels within the plastid [18,19]. Run-on analysis clearly demonstrates that the changes in mRNA abundance are largely due to transcript initiation [19].

This report presents data suggesting that the *Heterosigma akashiwo *response regulator component [20] of a His-to-Asp signal transduction circuit interacts with a nuclear-encoded sigma 70 (σ^70^) subunit of a eubacterial-like RNA polymerase to modulate chloroplast gene transcription. We propose that this simplified two-component/σ^70 ^– partnership found in *H. akashiwo *may offer insight to a mechanism by which chloroplast gene transcription is controlled in certain algal taxa.

## Results

### Tsg1 – a sensor kinase protein

The *Heterosigma akashiwo *chloroplast genome [21] contains a single His-to-Asp sensor kinase (transcriptional sensor gene 1, *tsg1*). The presence of a chloroplast encoded His-to-Asp sensor kinase gene is not universal in stramenopiles or other plastid-containing organisms (Table 1). A *tsg1 *homologue is found in the haptophyte *Emiliania huxleyi *(annotated as *dfr*) but not in the chloroplasts of the bacillariophytes *Odontella sinensis*, *Phaeodactylum tricornutum *and *Thalassiosira pseudonana*, the pelagophyte *Aureoumbra lagunensis*, the cryptophyte *Guillardia theta*, and the glaucophyte *Cyanophora paradoxa*. In rhodophytes, a *tsg1 *homologue is encoded in the chloroplasts of *Cyanidium caldarium *(*ycf26*), *Gracilaria tenuistipitata *var. *liui *(*dfr*), *Porphyra purpurea *(*ycf26*) and *P. yezoensis *(hypothetical chloroplast protein 26), but is absent in *Cyanidioschyzon merolae*. No *tsg1 *genes have been identified in any chlorophyte or charophyte examined to date.

**Table 1:** Distribution of sensor kinase and response regulatory proteins in photosynthetic plastids.

**Taxon/Organism**	**Sensor Kinase Copy Number**	**Sensor Kinase Accession Number**	**Response Regulator Copy Number**	**Response Regulator Accession Number**
**Raphidophyte**				
*Heterosigma akashiwo*	1	EF115378	2	CAB46638
**Pelagophyte**				
*Aureoumbra laguensis*	0	NP	0	NP
**Bacillariophyte**				
*Odontella sinensis*	0	NP	0	NP
*Phaeodactylum tricornutum*	0	NP	0	NP
*Thalassiosira pseudonana*	0	NP	0	NP
**Haptophyte**				
*Emiliania huxleyi*	1	YP_277392	1	YP_277359
**Cryptophyte**				
*Guillardia theta*	0	NP	1	NP_050679
**Glaucophyte**				
*Cyanophora paradoxa*	0	NP	2	NP_043139, NP_043243 (partial)
**Rhodophyte**				
*Cyanidioschyzon merolae*	0	NP	1	NP_849001
*Cyanidium caldarium*	1	AAF12904	2	Q9TLQ4, CAA44458
*Gracilaria tenuistipitata *var.*liui*	1	YP_063707	1	YP_063539
*Porphyra purpurea*	1	AAC08278	1	NP_053968
*Porphyra yezoensis*	1	YP_537073	1	YP_537039
*Porphyridium aerugineum*	--	NP	1	CAA44464
*Rhodella violacea*	--	NP	1	AAA62132
**Charophyte**				
*Chlorokybus atmophyticus*	0	NP	1	ABM87971

The dashed lines (--) indicate that the complete chloroplast genome for that taxon is not available, so the presence or absence of the gene is unknown. NP indicates not present.

**Figure 1:**
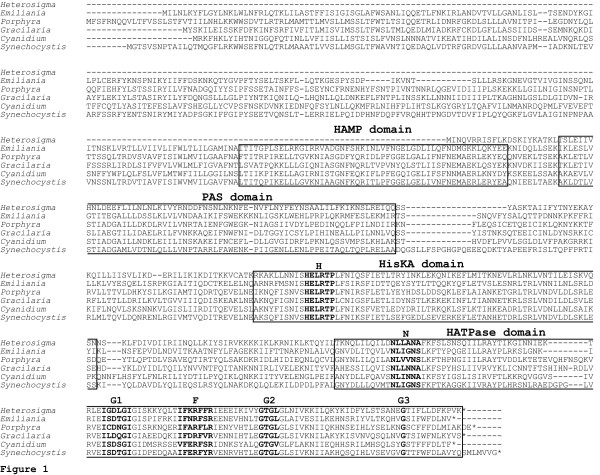
**Sequence comparison of *Heterosigma akashiwo *Tsg1 with putative homologues in rhodophytic plastids and a cyanobacterium**. Putative homologues of Tsg1 from *Heterosigma akashiwo *(accession number EF115378), *Emiliania huxleyi *(YP_277392), *Porphyra purpurea *(AAC08278), *Gracilaria tenuistipitata *var.*liui *(YP_063707), *Cyanidium caldarium *(AAF12904), and *Synechocystis *sp. PCC 6803 (BAA16687) are compared. The HAMP, PAS, HisKA, and HATPase domains are boxed. The H-box which contains the histidine residue, site of phosphorylation, N, G_1_, F, G_2_, and G_3 _sites are bolded.

The predicted amino acid sequence of *Heterosigma akashiwo *Tsg1 (43 kDa) has a shorter N terminus than that observed in rhodophytic algal sensor proteins and similar proteins found in cyanobacteria (Figure 1). Tsg1 lacks the transmembrane and HAMP domains [22] observed in some cyanobacterial and all chloroplast-encoded rhodophytic homologues (Figure 1), but it maintains the regions that contain the HisKA and HATPase domains. The Tsg1 protein has a weak PAS domain (Pfam e-value 5.4 and SMART [23,24] e-value 6.03 × 10^2^) that aligns with the well defined PAS domains of sensor kinases from *Porphyra purpurea, Gracilaria tenuistipitata *var. *liui*, *Cyanidium caldarium*, and *Synechocystis *sp. PCC 6803 (Figure 1).

The *Heterosigma akashiwo *Tsg1 kinase domain (Figure 1) displays a highly conserved H box (histidine phosphorylation site) whose consensus amino acid array HELRTP identifies this protein as a Type 1 (subtype B) histidine kinase [25]. Excellent sequence fidelity of the histidine kinase ATP binding site is also maintained for the N, G_1_, F and G_2 _motifs, including the conserved glycine G_3 _at its terminus [9,25,26]. The H to N distance between the histidine of the H box and the asparagine of the kinase domain is indicative of histidine kinase subtypes [25]. The 116 amino acid residues that lie between H and N in *H. akashiwo *Tsg1 and its homologues in the rhodophytic and chromophytic taxa are consistent with a Type 1 sensor protein identity. Nine lysine and three arginine residues contribute to the net positively charged amino acid sequence between the histidine kinase and ATPase domains in the *H. akashiwo *protein. Tsg1 of both *H. akashiwo *and rhodophytes shows close sequence similarity to the proteins Hik33 and NblS that serve as monitors of environmental stress in cyanobacteria [27,28].

### Trg1 – a winged helix-turn-helix protein

Two identical copies of *trg1 *were found on the chloroplast genome of *Heterosigma akashiwo*, one on each copy of the inverted repeat. The distribution of *trg1 *is varied among disparate taxa (Table 1). The *trg1 *gene has not been found in the pelagophyte *Aureoumbra lagunensis*. This gene is also missing from both the nuclear and chloroplast genomes of *Thalassiosira pseudonana *and *Phaeodactylum tricornutum *as well as the chloroplast genome of *Odontella sinensis*. A single copy of the *trg1 *gene has been identified in the chloroplast genomes of the haptophyte *Emiliania huxleyi *(*ycf27*), the cryptophyte *Guillardia theta *(*ycf27*), and the rhodophytes *Cyanidioschyzon merolae *(*ycf27*), *Gracilaria tenuistipitata *var. *liui *(*ompR*), *Porphyra purpurea *(*ycf27*), *P. yezoensis *(hypothetical chloroplast protein 27)*, Porphyridium aerugineum *(*ompR*) and *Rhodella violacea *(*orf246*; *ompR *homolog), whereas the glaucophyte *Cyanophora paradoxa*, has one full copy (*ycf27*) and a partial fragment of this gene (*orf27*). The rhodophyte *Cyanidium caldarium *chloroplast encodes two complete copies of *trg1 *(*ompR *and *ycf27*), though these genes are relatively divergent from each other (E-value 2.0 × 10^-78^). Interestingly, a *trg1*-like gene (*ycf27*) has been identified in the plastid genome of the charophyte *Chlorokybus atmophyticus*.

**Figure 2:**
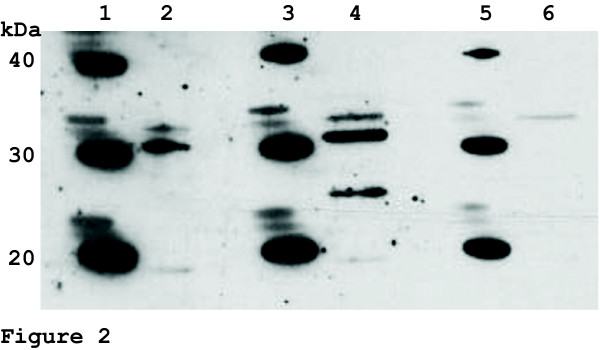
**Western blot showing the expression of Trg1 in *Heterosigma akashiwo***. Total soluble proteins were separated on NuPAGE Novex Bis-Tris gel and probed with preadsorption control (lane 2), anti-Trg1 peptide antiserum, day 50 (lane 4) and preinjection antiserum, day 0 (lane 6). A band of the expected estimated size of 27 kDa is present in the anti-Trg1 peptide lane 4, but not in the two negative controls (preadsorption and day 0). Lanes 1, 3, and 5 contain "Magic Mark Western" protein standard.

Western analysis was used to demonstrate that the Trg1 protein is expressed *in vivo*. Data shown in Figure 2 confirmed the presence of Trg1 protein in exponentially growing *Heterosigma akashiwo *cells that were harvested at L3 of a 12 h light:12 h dark growth cycle. An expected protein band of 27 kDa was present when cell extracts were exposed to post-bleed antiserum (Day 50; Figure 2 lane 4) and was absent in the lane exposed to preadsorption control (Figure 2 lane 2) and prebleed antiserum (Figure 2 lane 6). These data confirmed that the low abundance message for the *trg1 *gene [20] is translated into a protein product.

**Figure 3:**
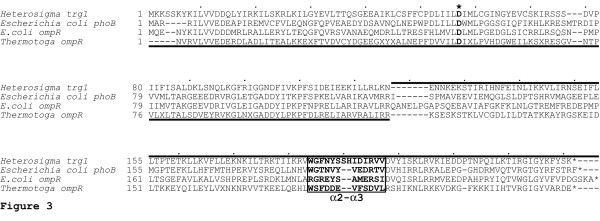
**Sequence comparison of *Heterosigma akashiwo *Trg1 with select bacterial response regulators**. The *Heterosigma akashiwo *Trg1 sequence (accession number CAB46638) was aligned with *Escherichia coli *PhoB (P03025), *E. coli *OmpR (P08402) and *Thermotoga maritima *OmpR (1KGSA). Receiver and DNA binding domains are underlined and overlined, respectively. Within the receiver domain, the phosphorylation site is indicated by an asterisk (*). Within the DNA binding domain, the RNA polymerase binding site (α2-α3 loop) is boxed and bolded.

Response-regulatory proteins, such as Trg1, contain a receiver domain and a DNA recognition domain. The DNA recognition domain affects gene expression by interacting with both DNA and with RNA polymerase [29,30]. The inferred amino acid sequence of *Heterosigma akashiwo *Trg1 was compared with Trg1-like sequences from *Thermotoga maritima *(OmpR) and *Escherichia coli *(OmpR and PhoB) for which tertiary structures have been determined [30-33]. A number of conserved regions are shared among these sequences both within the receiver domain and DNA binding domain (Figure 3).

**Figure 4:**
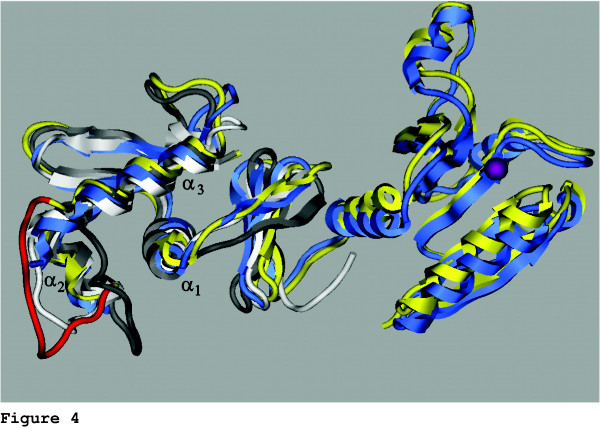
**Molecular model of *Heterosigma akashiwo *Trg1**. Structural model of the DNA recognition domain of Trg1 (shown in yellow) based on the defined partial structures of *Escherichia coli *OmpR (grey), PhoB (white), and the complete receiver-regulator structure from *Thermotoga maritima *(blue) reveals important similarities. The predicted Trg1 model closely resembles that of OmpR, particularly in the putative DNA binding region (α3 helix). Notably, the predicted Trg1 structure for the putative RNA polymerase interaction site (α_2_-α_3 _loop, red) more closely matches that of PhoB. The phosphorylation site is shown as a purple sphere.

Comparison of *Heterosigma akashiwo *Trg1 and *Thermotoga maritima *OmpR shows good three-dimensional similarity (Figure 4). To gain insight into *H. akashiwo *Trg1-RNA polymerase structure/function relationship as a possible transcriptional regulator of chloroplast genes, we constructed a model of the DNA binding domain for this protein. Because *T. maritima *lacks the α_2_-α_3 _polymerase loop, our model of this region is based on available three-dimensional structures for OmpR and PhoB. OmpR has an X-ray generated structure [32] at 1.95 Å resolution (10 PC) whereas the PhoB report represents an NMR (1QQI) derived structure [30]. Structures of OmpR and PhoB have been previously compared, and our observations are based upon that comparison [31]. The most notable differences between OmpR and PhoB occur in the putative eubacterial-like RNA polymerase contact loop (α2-α3 region) and at the loop between helix α3 and strand β5. The higher sequence similarity between Trg1 and PhoB in the α2-α3 region leads us to believe that PhoB is a better structure model for residues 130–208 and similarly OmpR for residues 209–231 (Figures 3 and 4). Thus, we constructed a chimeric model from these two portions, which yielded satisfactory internal and external environments for most residues.

Mutational analysis of the PhoB "RNA polymerase loop" region shows that the amino acids W184, G185, V190 and D192 are needed for successful association of this response regulator protein with the σ^70 ^subunit of prokaryotic RNA polymerase [30]. The first two of these amino acids are present in Trg1 (as is V183) but V190 is conservatively replaced by I192, and the D192 has been replaced with a non-conservative I194 (Figure 3). Similar to observations in *Heterosigma akashiwo *Trg1, conservation of a PhoB-like domain is found within the response regulator α_2_-α_3 _loops of some cyanobacteria and proteobacteria, as well as in chloroplasts (Table 2). The observation that the *H. akashiwo *Trg1 α_2_-α_3 _loop is two amino acids longer than that seen in PhoB and OmpR suggests that either the response regulator may interact with a undescribed RNA polymerase subunit or the RNA polymerase subunit itself may also be modified.

**Table 2:** Comparison of putative RNA polymerase association loop domain among prokaryotic and plastid-encoded response regulators.

**Organism**	**Gene Name**	**Taxon**	**Putative RNA Polymerase Loop Domain**	**Accession Number**
***Heterosigma akashiwo***	***trg1***	**raphidophyte**	**WGFNYSSHIDIRVV**	CAB46638
*Emiliania huxleyi*	*ycf27*	haptophyte	WGYTPERYLDTRVV	AAX13858
*Guillardia theta*	*ycf27*	cryptophyte	WGYTPERHVDTRVV	AAC35613
*Cyanophora paradoxa*	*ycf27*	glaucophyte	WGYTPERHIDTRVV	AAA81319
*Cyanidioschyzon merolae*	*ycf27*	rhodophyte	WGYAWPNETRVV	BAC76163
*Cyanidium caldarium*	*ycf27*	rhodophyte	WGYKLSKHEPIADTRIV	AAF12879
*Cyanidium caldarium*	*ompR*	rhodophyte	WGYTPERHLDTRVV	CAA44458
*Gracilaria tenuistipitata var. liui*	*ompR*	rhodophyte	WGYKPERHVDTRVV	YP_063539
*Porphyra purpurea*	*ycf27*	rhodophyte	WGYTPERHVDTRVV	AAC08244
*Porphyra yezoensis*	*ycf27*	rhodophyte	WGYTPERHVDTRVV	Q1XDC9
*Porphyridium aerugineum*	*ompR*	rhodophyte	WGYTAERQVDTRVV	CAA44464
*Rhodella violacea*	*ompR*	rhodophyte	WGYTPERHIDTRVV	AAA62132
*Chlorokybus atmophyticus*	*ycf27*	charophyte	WEYGNDSYIDTRVV	ABM87971
*Nostoc *sp. PCC7120	*ycf27*	cyanophyte	WGYTPERHVDTRVV	BAB75521
*Synechocystis *sp. PCC6803	*ycf27*	cyanophyte	WGYTPERHVDTRVV	BAA18408
*Tolypothrix *sp. PCC7601	*rpaB*	cyanophyte	WGYTPERHVDTRVV	AAD30119
*Bacillus subtilis*	*recM*	Firmicutes	WGYDYFGDVRTV	BAA05173
*Clostridium petringens*	*ompR*	Firmicutes	WGYEYIGETRTV	BAB80348
*Lactobacillus sakei*	*rrp3*	Firmicutes	WGYDYFGDVRTV	AAD10263
*Listeria innocua*	*ompR*	Firmicutes	WGYDYFGDVRTV	CAC95548
*Streptomyces coelicolor*	*scrA*	Firmicutes	WGYRHAADTRLV	AAG15433
*Thermotoga maritima*	*drrA*	Thermotogales	WGYDYYGDTRTV	AAD36722
*Ralstonia metallidurans*	*czcR*	Proteobacteria	WGVNFDTDTNVV	CAA67086
***Escherichia coli***	***phoB***	**Proteobacteria**	**WGTNVYVEDRTV**	P03025
***Escherichia coli***	***ompR***	**Proteobacteria**	**RGREYSAMERSI**	P08402

The putative RNA polymerase association loop domain was identified in prokaryotic and plastid-encoded response regulators based on molecular modelling for Trg1, PhoB, and OmpR [31]. For easy comparison in the table – the data for Trg1, PhoB, and OmpR are bolded.

### Presence of *rpoD *in *Heterosigma akashiwo*

**Figure 5:**
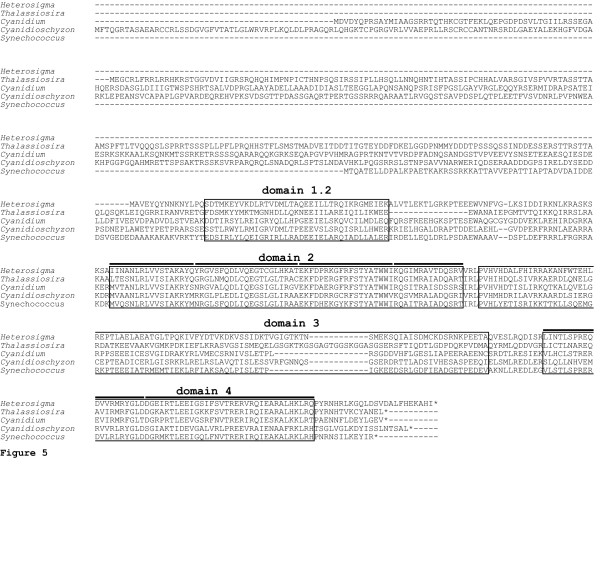
**Sequence comparison of *Heterosigma akashiwo *RpoD with putative homologues in chromophytic and rhodophytic algae, and a cyanobacterium**. Putative homologues of RpoD from *Heterosigma akashiwo *(accession number EF115377), *Thalassiosira pseudonana *[43], *Cyanidium caldarium *(BAA11832), *Cyanidioschyzon merolae *[102], and *Synechococcus *sp. PCC 7942 (BAA01749) are compared. Conserved domains (1.2, 2, 3, and 4) are boxed and sub-domains (2.1–2.4, 4.1, and 4.2) are identified as bars above their respective boxes.

Data presented above support the hypothesis that *Heterosigma akashiwo *Trg1 interacts with a σ^70^-like subunit of a eubacterial-like RNA polymerase. Sequence analysis of *H. akashiwo *revealed the presence of a nuclear-encoded *rpoD *gene. The presence of both a signal peptide sequence and a putative stromal targeting domain on the amino terminus of the RpoD protein supports the hypothesis that this protein is chloroplast-targeted (Table 3). Although *H. akashiwo *RpoD lacks the autoinhibitory 1.1 region, it retains the highly conserved functional domains (1.2, 2.1 – 2.4, 3.0 – 3.2 as well as 4.1 and 4.3) that have been elucidated for eubacterial homologues ([34]; Figure 5). Most striking in the context of this study is the extensive maintenance of sequence identity within domains 4.1 and 4.2 among phylogenetically diverse organisms (Figure 5). This domain is responsible for interaction with transcriptional regulator proteins and with the -35 promotor array [34-36].

**Table 3:** *Heterosigma akashiwo *signal peptides

**Precursor Protein**	**Accession Number**	**Signal peptide**	**Beginning of STD**
RpoD	EF115377	VAHCTTFAYKGSNMRSHFFFMLWSVSVAATAA	**F**MMPGR...
Fcp1	X99697	MSLKLATLAAALMGASA	**F**VAPNKM...
Fcp2	EF115376	VVFDLYASTIPSVNQAHKSKMSLKLATFAAALAGASA	**F**VAPNQM...
PRK	EF115375	MMYKLATLLALLPAVVA	**F**TTSFNG...
PsbO	AY130990	MKFVAVLVCLMVSAVVA	**F**KTQRN...

*Heterosigma akashiwo *RNA polymerase D subunit (RpoD), fucoxanthin chlorophyll binding protein 1 (Fcp1), fucoxanthin chlorophyll binding protein 2 (Fcp2), phosphoribulose kinase (PRK), and 33 kDa oxygen-enhancer 1 protein (PsbO) precursors showing the signal peptide and the start of stromal targeting domain (STD). The conserved phenalynine (F) indicating the beginning of stromal targeting domain [62, 97, 98] is marked in bold.

Phylogenetic analyses of *rpoD*/σ^70 ^have been published for some eubacteria [4,37-40], and land plants [41,42]. We attempted to generate a phylogeny to place *Heterosigma akashiwo rpoD *among other photosynthetic eukaryotes (bacillariophytes, cryptophytes, glaucophytes, rhodophytes, chlorophytes, and charophytes). Since this comparison yielded few regions that could be confidently aligned, the resulting trees were consistently poorly supported as measured by bootstrap values and posterior probabilities (results not shown). A consistent phylogenetic result, however, was the placement of *H. akashiwo rpoD *as sister to one of the six *rpoD*/*Sig *sequences that had been annotated from the complete nuclear genome of *Thalassiosira pseudonana *[43].

A number of problems common in phylogenetic inference could explain our difficulty in generating a robust phylogeny. These include problems associated with parology, lineage sorting and horizontal gene transfer [for discussion see [44,45]], long-branch attraction [46], and rate heterogeneity among lineages [47].

## Discussion

Most plausibly, the prokaryotic ancestor(s) of eukaryotic chloroplasts were fully equipped with their own multifaceted signal transduction circuitry [14,48], as observed in cyanobacterial and proteobacterial genomes which encode multiple sensor kinase/response regulator pairs [11,12,49]. Since the chloroplast must respond to both extra- and intra-cellular cues, one might anticipate strict conservation of these signal transduction arrays. However, given the observations that fewer sensor/response circuits exist in intracellular bacterial pathogens than in free-living representatives [50], one may argue that a reduction in the ancestral plastid genome size after endosymbiosis may have driven the loss of chloroplast-encoded His-to-Asp regulatory arrays (see Table 1).

The chloroplast genomes (Table 1) of several rhodophytic algae [51-56], the glaucophyte *Cyanophora paradoxa *[57], the haptophyte *Emiliania huxleyi *[58], the cryptophyte *Guillardia theta *[59], and the charophyte *Chlorokybus atmophyticus *[60] have been shown to encode the response regulator gene and in some, the sensor kinase gene for the His-to-Asp proteins. The presumptive loss of the sensor kinase seen in some chloroplast genomes may suggest that under such a circumstance, the regulatory protein may be governed by nuclear-encoded sensor kinases or by yet undescribed accessory proteins that are either of nuclear or chloroplast origin [see [9] for discussion]. The occurrence of this signalling array is less well documented in stramenopiles. The chloroplast genome of the raphidophyte *Heterosigma akashiwo *encodes a single response regulator and its cognate sensor kinase [[20] and this study], but the chloroplast genomes of representatives within the bacillariophytes and the pelagophytes lack both proteins of this two-component system [61,62]. More than 75 green plant (~9 chlorophyte and ~66 charophyte) chloroplast genomes have been sequenced. Of these only the charophyte *Chlorokybus atmophyticus *encodes a response regulator protein. It should be noted, however, that partial footprints of (laterally transferred?) prokaryotic His-to-Asp transduction pairs have been identified in some green-plant nuclear genomes [63-66]. Such truncated His-to-Asp constructs have been shown to signal mitogen-activated protein kinase cascades, which cause the differential regulation of targeted genes [63,67].

The taxonomic distribution of this gene-pair is poorly understood. Complete chloroplast genomes are few, especially in species-rich lineages that are represented by only a small number of complete chloroplast genomes (e.g., the bacillariophytes). Whether the maintenance of the His-to-Asp signal transduction apparatus in the chloroplast corresponds to established phylogenies remains to be determined. One might anticipate that the chloroplasts of chromophytic algae would retain this His-to-Asp array since they are the product of a serial endosymbiotic event, which involved a rhodophytic algal ancestor. Unfortunately, data for the diverse taxonomic assemblage of chromophytes has been both minimal and conflicting. The presence of the His-to-Asp array in *Heterosigma akashiwo *appears to reflect the retention of an ancestral signature. Whether the loss of the His-to-Asp pair in bacillariophyte chloroplast DNA represents a derived genotype, remains an open question. Regardless of phylogenetic profile, the evolutionary retention of all or part of a His-to-Asp signal transduction circuit in some distantly related algal chloroplasts strongly suggests that this biochemical mechanism must play an important role in the maintenance of chloroplast homeostasis.

We propose that the *Heterosigma akashiwo *Tsg1/Trg1 signal transduction pair, in concert with an RNA polymerase σ^70 ^subunit, is involved in regulating chloroplast gene transcription. The environmental stimulus that regulates the signal transduction response remains elusive. The inability to create gene-knockout mutants or perform transformation experiments in chromophytic algae (except diatoms, which lack His/Asp genes) has hampered gene expression studies that are needed to provide direct evidence for the role of His/Asp systems in chloroplast function. However, one might infer function given the similarity of the chloroplast-encoded *H. akashiwo *Tsg1 and the *ycf26*-encoded proteins of *Emiliania huxleyi*, *Cyanidium caldarium*, *Gracilaria tenuistipitata *var. *liui*, *Porphyra purpurea *and *P. yezoensis *to the cyanobacterial sensor kinases and NblS. Hik33 has been shown by deletion studies to impact the expression of selected genes in response to osmotic and low temperature stress [27,68,69], while its homologue NblS is reported to serve as a sensor of nutrient stress and high light intensity [28]. The underlying mechanism driving these physiological responses may be governed by redox and light signals for two reasons [discussed in [27,28]]: (a) both Hik33 and NblS possess a PAS domain, which is thought to be involved in redox and light sensing [70] and, (b) a large majority of the genes impacted by Hik33 and NblS are related to photosynthesis [27,28]. It has been proposed that redox control of gene expression is a fundamental evolutionary selection mechanism responsible for the maintenance of chloroplast-encoded gene regulation systems [71,72]. As shown in Figure 1, the *H. akashiwo *Tsg1 protein has a putative PAS domain. While the three dimensional structure of PAS domains is conserved among taxa, the primary protein sequences that comprise this motif are often diverse [73]. As more types of PAS domains are characterized, the E-value for the PAS domain in *H. akashiwo *Tsg1 should become more robust.

An important difference between *Heterosigma akashiwo *Tsg1 and its homologues is the absence of a transmembrane region and a HAMP domain. The possibility of a split *tsg1 *gene was not supported by detailed analysis of the completely sequenced *H. akashiwo *chloroplast genome. The absence of a transmembrane region implies that Tsg1 is most likely present in the stroma. Though the majority of described Tsg1 proteins are putatively membrane bound (data not shown, SMART database search [23,24]), soluble histidine kinases have been identified [74,75]. Studies are underway using a Tsg1 peptide antibody to verify the location of the protein in the cell.

Contrary to a previous report [76], our data indicate that RpaB in the cyanobacterium *Synechocystis *shares not only similarity to the ycf27 proteins in red algae but also to *H. akashiwo *Trg1. The premise that ycf27 homologues are restricted to eukaryotic algae containing phycobilisomes [76,77] is contrary to the description of this protein in the non-phycobilisome containing algae – *Heterosigma akashiwo *(Trg1) [20], *Guillardia theta *(ycf27) [59], *Emiliania huxleyi *(ycf27) [58], and *Chlorokybus atmophyticus *(ycf27)[60]. Nonetheless, the hypothesis that RpaB regulates the synthesis of (unknown) "factors required to couple phycobilisomes to PS1 or PSII" [76,77] is consistent with the possible role of this protein in redox/light sensing.

The assignment of *Heterosigma akashiwo *Trg1 to the "Class 2" (or "*ompR*" super family) of transcriptional regulators offers additional insight to its function in the plastid. In prokaryotic cells, some "Class 2" proteins (such as PhoB) regulate transcription through interaction with the σ subunit of RNA polymerase [78] while others associate with the α subunit of this enzyme [29,79]. We have identified a PhoB-like signature for the RNA polymerase recognition domain in Trg1 and a putative chloroplast-targeted σ^70 ^subunit in *H. akashiwo*. A comparable transcriptional mechanism appears to be present in *Cyanidioschyzon merolae*, *Cyanidium caldarium*, and *Guillardia theta *as both PhoB-like signatures and σ subunits have been identified (Table 2) [55,80-83].

Sigma factors in concert with core RNA polymerase selectively target chloroplast genes for transcription. For example, the prokaryotic-like, plastid-encoded polymerases with their associated σ factor(s) exclusively transcribe many genes that impact the photosynthetic process including *rbcL*, *psbA*, *psbD*, *petB*, *ndhA*, *atpI*, *atpH *and *rps14 *[84-87]. Both plastid-encoded polymerase and the phage-like nuclear-encoded polymerase can transcribe *rrnA*, *atpB*, *clpP*. It should be noted however, that these eubacterial RNA polymerase-associated σ factors often interact with regulatory proteins (such as Trg1) and this association may further influence the transcription of specific genes [3].

How could the transcription of a small, select set of genes impact chloroplast homeostasis? One might propose that a hierarchical assembly of proteins during the formation of molecular complexes could provide an exceptionally efficient mechanism for regulating the quantitative and qualitative production of molecular structures necessary for the maintenance of chloroplast function. In the chloroplast, many large functional complexes that drive oxygenic photosynthesis and carbon fixation are constructed with a definitive stoichiometry that reflects the cooperative interaction between plastid and nuclear genomes. Studies suggest that protein complex formation is regulated by the presence of a "dominant assembly partner" whose presence assures the production of its assembly associates in the correct proportions [88]. For example, a *Chlamydomonas *mutant lacking the D1 protein (encoded by *psbA*) expresses only minimal levels of D2 (*psbB*) as well as CP47 (*psbC*) proteins. Similarly, mutants in CP47 (*psbC*) or D2 (*psbB*) show depressed concentrations of D1 (*psbA*) protein. In contrast, *Chlamydomonas *cells that were mutated in CP43 (*psbD*) were able to assemble D1, D2 and CP47 into a stable complex. The existence of such an assembly cascade is not restricted to photosystem II construction. When cytochrome b6 (*petB*) or subunit IV (*petD*) are not present, cytochrome f (*petA*) synthesis drops to 10% of that in wild type cells. A similar synthesis cascade appears also to occur in the biogenesis of ATP synthetase, which is comprised of five subunits (α,β,γ,δ,ε). Mutants lacking β will not accumulate any other core peptide. In effect, a minimal signal transduction system (encoded in the chloroplast) in conjunction with the σ subunit (encoded in the nucleus) may have given the ancestral eukaryotic cell a simple and efficient method to integrate chimeric gene sets.

## Conclusion

We have identified a His-to-Asp signal transduction array in the secondary endosymbiotic chloroplast of the stramenopile *Heterosigma akashiwo*. These proteins are similar to those found in bacteria and in the chloroplast genomes of several, though not all, algae.

This study generated a number of interesting questions. For example, why have several of the red lineage chloroplasts retained all or part of the His-to-Asp signal transduction system while only a single green chloroplast lineage retained this system? Will the sensor kinase protein be more frequently missing from this pair as additional stramenopile chloroplast genomes are analyzed? Are there undescribed chloroplast-encoded proteins that can substitute for sensor kinases? Are all sensor kinases triggered by the same or by different environmental cues? What are those cues? When more than one RpoD protein is present in an organism (e.g., *Thalassiosira*), does one member of this σ^70 ^factor family interact with the regulator protein or do different sigma/response regulators form partnerships that control the expression of specific gene sets? With the advent of high throughput genome sequencing, more effective bioinformatics/evolutionary analysis as well as extensive molecular studies of cellular processes are now possible. From the questions posed above, it is evident that events associated with chloroplast gene regulation will continue to provide a challenging field for future research.

## Methods

### Cell culture

All algal cultures were maintained on a 12 hr light:12 hr dark (diel) photoperiod using cool white light (60 to 80 μEm^-2^s^-1^) with continuous rotary shaking at 60 rpm. *Heterosigma akashiwo *(Hada) Hada ex Hara et Chihara (strain CCMP-452) was originally obtained from Sarah Gibbs (McGill University, Canada). One liter vegetative cultures were axenically maintained on an artificial sea water medium as previously described [20,89]. Cells were counted using a Coulter counter (model Z2 Coulter Particle Count and Size Analyzer; Beckman Coulter, Fullerton, CA, USA) equipped with a 100 × 120 μm aperture.

### RNA isolation

Axenic *Heterosigma akashiwo *cultures were harvested at a density of 5 × 10^5 ^cells/mL and a modified protocol [89] was used to obtain total RNA from these cells. All steps were carried out with chilled reagents and performed at 4°C. *H. akashiwo *cells were collected by centrifugation at 1,000 × g for 5 min. Pelleted cells were resuspended to a concentration of 2 × 10^7 ^cells/mL in a pH 7.5 buffer that contained 25 mM KCl, 25 mM MgCl_2_, and 25 mM Tris (KMT buffer). KMT-saturated phenol was added to the lysed cell mixture at a ratio of (1.5:1). The phenol:supernatant mixture was inverted slowly using a rotating wheel for 20 min, followed by centrifugation at 9,750 × g for 15 min in a fixed angled rotor. An equal volume of KMT-saturated phenol:chloroform:isoamyl alcohol (75:24:1) was added to the retrieved supernatant and the inversion-centrifugation steps repeated. The supernatant was removed and extracted twice more as described above but with chloroform:isoamyl alcohol (24:1) at a 1:1 ratio. RNA was precipitated by adding 2 volumes of -20°C ethanol (95%) and chilling overnight at -20°C after which it was pelleted at 9,750 × g for 15 min. The ethanol was decanted and the RNA was air dried at room temperature. One mL of RNase free water was added to the RNA and left to dissolve on ice for 15 min. To remove DNA contamination from total RNA used for RACE, 40 μg of total RNA was incubated with 10 U of DNase I in a 100 μL reaction volume according to the manufacturer's instructions (Amplification Grade, Invitrogen, Carlsbad, CA, USA). After the 15 min incubation period with DNase I, total RNA was purified using the Qiagen RNeasy kit (Qiagen, Valencia, CA, USA) following the RNeasy Mini Protocol for RNA cleanup. The removal of DNA contaminants was monitored by visualizing purified RNA on a 1.5% agarose gel containing 2.2 M formaldehyde and 1X MOPS [90].

### Amplification of 550 bp from *rpoD*

Primers were designed to conserved regions of the *rpoD *gene by aligning σ factors from *Cyanidium caldarium *SigC(accession number BAA25788), *Guillardia theta *(BAB87262) and *Thalassiosira pseudonana *[43]. PCR amplification of the partial *rpoD *fragment was carried out in a 50 μL reaction that contained 100 ng of template DNA, 10 pmole of each primer ORAC 1104 and ORAC 1105 (Table 4), 1.5 Units of *Taq *Polymerase, 1X *Taq *polymerase buffer, 1.5 mM MgCl_2 _(Promega, Madison, WI, USA), and 0.2 mM of each dNTP (Invitrogen). Initial denaturation of DNA was at 94°C for 3 min followed by 30 cycles of 94°C for 1 min, 50–60°C for 1 min, and 72°C for 1 min. A final extension was carried out at 72°C for 7 min. The PCR product was separated on a 1% TBE agarose gel and the dominant 550 bp fragment purified using the QIAquick Gel Extraction Kit (Qiagen). The purified 550 bp product was sequenced at the University of Washington Biochemistry Sequencing Facility using ABI 3730XL high-throughput capillary DNA Analyser (Applied Biosystems, Forest City, CA, USA).

**Table 4:** Primers used for PCR, RACE, and genome-walking to generate *rpoD *and *tsg1 *gene sequences.

**Primer name**	**Sequence**
PCR	
M13 forward	5'-GTAAAACGACGGCCAG-3'
M13 reverse	5'-CAGGAAACAGCTATGAC-3'
ORAC 1104	5'-AGGCTGACGAAGCTTGTGCAA-3'
ORAC 1105	5'-TTCTCTACCTACGCAACATGGTGG-3'
	
RACE	
ORAC 240	5'-GATACGGTGAAGGACAAGGTCTCATC-3'
ORAC 242	5'-CAGTGGCTTCTGCAAGCTCCGCGAG-3'
	
Genome walking	
ORAC 231	5'-TACCCAAAGTCTTCTCCAGTGTAACTAGAG-3'
ORAC 230	5'-GAATTTCTTCTTGTGCCGTCAACATATCG-3'

### 5' and 3' *rpoD *RACE

BD SMART RACE cDNA amplification kit (BD Biosciences Clontech, Palo Alto, CA, USA) was used to extend the known *rpoD *sequence. 5' and 3' RACE primers were designed using the sequence generated from the amplification of 550 bp *rpoD *fragment by PCR (see above). Total RNA was used to synthesize 5' RACE cDNA using 5'-CDS primer and BD SMART II A oligo primer and the 3' RACE cDNA using 3'-RACE CDS Primer A according to the manufacturer's instructions. A negative control to verify the absence of genomic DNA was performed by the exclusion of the reverse transcriptase. The 5' RACE cDNA and negative control were amplified with Advantage 2 PCR kit using the universal primer mix (UPM) from the SMART-RACE kit and ORAC 242 primer (Table 4). 3' RACE cDNA and negative control were amplified using UPM and ORAC 240 primers (Table 4). PCR protocol included an initial 3 min denaturation at 94°C, and then 30 cycles at 94°C for 30 sec, 60°C for 30 sec, and 72°C for 1 min. At the end of these cycles an additional 5 min at 72°C was performed to complete DNA synthesis. PCR products were purified from the reaction mixture using the QIAquick PCR purification kit and cloned into pCR-Blunt II-TOPO vector (Invitrogen). Randomly selected clones grown on Luria broth kanamycin (25 μg/mL) plates were checked for inserts using PCR. To screen for inserts, 20 μL of the 5 mL overnight cultures (25 μg/mL kanamycin in Luria broth) were centrifuged at 16,000 × g for 5 min and each resuspended separately in 100 μL of ddH_2_0. For the PCR, 5 μL of the cell suspension was added to a 50 μL volume reaction that contained 1X PCR buffer for KOD Hot Start DNA polymerase (EMD Biosciences, Novagen Brand, Madison, WI, USA), 0.2 mM dNTP, 1 mM MgSO_4_, 20 pmole of M13 forward and reverse primers (Table 4), and 1 U KOD Hot Start DNA polymerase. Plasmid DNA containing inserts was isolated using Qiaprep spin Miniprep columns (Qiagen) according to the manufacturer's directions. The inserts sequences were determined by dye-terminator automated sequencing using M13 forward and reverse primers (SigmaGenosys, The Woodlands, TX, USA) on 5 clones each for both the 5' RACE and 3' RACE.

### Genome walking to obtain the 5' UTR region of the *rpoD *gene

High molecular weight DNA was extracted from *Heterosigma akashiwo *cells grown to a density of 1.3 × 10^5 ^cells/mL. Harvested cells were extracted by following an on-line protocol [91]. The cells (8.7 × 10^7^) were resuspended in 20 ml of cold lysis buffer (20 mM EDTA, 10 mM TrisCl, pH 8, 1% Triton X, 500 mM Guanidine-HCl, and 200 mM NaCl). The lysate was incubated at 37°C for 1 hour with gentle agitation. The DNA was treated with RNAse A (20 μg/mL) for 30 min at 37°C followed by Proteinase K (0.8 mg/mL) for 2 h at 50°C using gentle agitation. To remove cell debris, this lysate was pelleted by centrifugation at 9,750 × g for 20 min and the clear supernatant was removed. Three mL of the lysate were added to each QBT buffer equilibrated Qiagen genomic 100 tip. The columns were washed twice with 10 mL of buffer QC. DNA was eluted from the genomic 100 tip with 5 mL of buffer QF and precipitated by the addition of 0.7 volume room temperature isopropanol. The DNA was pelleted by centrifugation at 9,750 × g for 20 min and the air-dried pellet resuspended in 1 mL of buffer EB (Qiagen). The DNA was stored at 4°C to prevent shearing from freeze thawing. The BD GenomeWalker™ Universal Kit (Clontech) was used to make four libraries by digesting the high molecular weight DNA with *DraI*, *EcoRV*, *PvuII*, and *StuI*, followed by DNA purification, and ligation of genomic DNA to BD GenomeWalker™ adaptors according to the manufacturer's instructions. The Genome Walker libraries were used to amplify DNA upstream of the known *rpoD *sequence by using ORAC 233 primer (Table 4) and AP1 primer provided in the kit by using BD Advantage 2 Polymerase Mix according to BD GenomeWalker™ Universal procedures. A nested PCR was performed using primer ORAC 232 (Table 4) and AP2 primer (kit primer) as suggested in the manual. The PCR products were separated on a 1% TAE gel and the dominant bands were excised and purified using the Qiagen Gel extraction protocol. The purified products were cloned into TOPO TA vector (Invitrogen) and sequenced using M13 forward and reverse primers.

### Domain analysis of Tsg1 protein and its homologues in a cyanobacterium and in rhodophytes

Similarity searches for Tsg1 were performed using the NCBI protein-protein BLAST algorithm [92]. Domain architecture analysis was carried out for the four proteins of Figure 1 using the SMART database default parameters. Amino acid sequences were aligned using Clustal W version 1.83 [93] as implemented by GenomeNet Computation Service [94] with default parameters. The domains from the SMART dataset were then used to establish the boundaries for the PAS, HAMP, HisKA and HATPase domains for Tsg1 in the Clustal W alignment. It was unnecessary to make further refinements since the domains fell into groups and few sequence gaps were present.

### Peptide signalling and stromal targeting domains within *rpoD *transcript

The *rpoD *sequence, and unpublished data generated in our laboratory for fucoxanthin chlorophyll binding protein 2, and phosphoribulose kinase transcripts were submitted to SignalP 3.0 Server [95,96] for identification of the signal peptide. The beginning of the stromal targeting domain was designated by the presence of a conserved phenylalanine residue [62,97,98].

### Trg1 peptide antibody and Western detection of Trg1

An oligopeptide (amino acid sequence-CDEIEEKILLRLKNENNKEK) unique to the *Heterosigma akashiwo *Trg1 sensor kinase was synthesized based on a hydrophilic region in the amino terminal end of the Trg1 protein. Synthesis and purification (85%) of this oligopeptide was done at Lampire Biologicals (Pipersville, PA, USA). The synthesized peptide was coupled to the carrier protein keyhole limpet hemocyanin (KLH) through the cysteine residue at the N-terminus of the peptide. Polyclonal antibodies (IgG) were directed against Trg1 peptide in three rabbits; each rabbit injected with 0.5 mg of purified peptide in Freund's complete adjuvant. Pre-immune antisera, collected prior to the initial injection, and total purified antisera (day 50), were both supplied by Lampire Biologicals and stored at -80°C until use.

Western blots were performed using X-Cell Sure Lock Mini Cell and NuPAGE MOPS SDS buffer kit (Invitrogen) according to manufacturer's instructions. *Heterosigma akashiwo *cells (1.6 × 10^6 ^cells) were harvested at a density of 1.3 × 10^5 ^cells/mL at L3 in a 12 h light:12 h dark period. The cells were pelleted at 3,800 × g for 10 min and the supernatant decanted. The pelleted cells were resuspended in 200 μL of NuPAGE lysis buffer and heated to 70°C for 10 min. The supernatant was clarified by centrifuging the lysed cells at 10,200 × g for 5 min at room temperature. The clarified protein samples (10 μL) and 2 μL of "Magic Mark Western" protein standard (Invitrogen) were separated on a NuPAGE Novex Bis-Tris gel (Invitrogen) at 180 V for 1 hr at 20°C. Proteins were electrophoretically transferred to BioRAD Transblot nitrocellulose membrane (0.45 μm, BioRAD Laboratories, Hercules, CA, USA) in NuPAGE transfer buffer with the X-Cell Blot Module for 1 hr at 30 V at 20°C. The blot was blocked at 4°C overnight using blocking buffer (1X PBS, pH 7.0, 3% non-fat dried milk, and 0.05% Tween-20). A preadsorption control was carried out to establish the specificity of the polyclonal antibodies to Trg1 by exposing the postbleed serum (10 μL) to 2 mg of Trg1 oligopeptide overnight at 4°C. After the overnight incubation, the preadsorption control was centrifuged at 16,000 × g for 20 min and the supernatant removed. Blots were probed with prebleed serum, postbleed serum, and the preadsorption control at a dilution of 1:500 for 1 hr at room temperature. The membranes were treated in wash buffer (10 mM Tris-HCl, pH 8.0, 150 mM NaCl, and 0.05% Tween-20) with four buffer changes of 45 min each. The Super Signal West Pico anti-rabbit IgG detection kit (Pierce, Rockford, IL, USA) was used to detect Trg1 protein. The blots were gently agitated at room temperature while submersed in the diluted secondary antibody (1:50,000). Four washes (45 min each) in wash buffer were used to remove the unbound secondary antibody. After washing, 3 mL of the working solution from the Pierce kit (equal parts of stable peroxide solution and luminal/enhancer) were combined and placed on the membranes. The blots were placed on CL-Xposure film (Pierce) and developed in Fischer Model K-Plus Automatic X-Ray film processor (Fischer Industries Inc., Geneva, IL, USA) after 4 min.

### Trg1 protein modelling

Three know structures were used to model *Heterosigma akashiwo *Trg1: *Thermotoga maritima *OmpR (1 KGS), *Escherichia coli *PhoB (1QQI), *E. coli *OmpR (1OPC) [30-33]. Structures were obtained from the Protein Data Bank [99,100]. Modelling was carried out with tools available in the suite of programs in the "Molecular Operating Environment" from the Chemical Computing Group, Montreal [101].

## Abbreviations

*tsg1 *– transcriptional sensor gene 1; *trg1 *– transcriptional regulator gene 1; *rpoD *– RNA polymerase D subunit; CCMP – Provasoli-Guillard National Center for Culture of Marine Phytoplankton

## Authors' contributions

MRD designed the Trg1 antibody and carried out Western analyses, performed RACE and genome walking experiments for *fcp2 *and *rpoD*, performed domain analysis and alignment of Tsg1 and its homologues, analysed signal peptides and stromal targeting domains for RpoD, Fcp2, and PRK, and contributed to the manuscript (text and figures). KGK constructed RNA polymerase alignments, performed phylogenetic analyses, and contributed to the manuscript (text and figures). ETA carried out modelling of the Trg1 protein and contributed to the final manuscript (text and figures). LYSC amplified and sequenced the 550 bp RNA polymerase gene fragment and assisted in the construction of the final *rpoD *sequence. MAJ sequenced *tsg1*, participated in modelling of the Trg1 protein and contributed to the development of the manuscript. RAC conceived of the study, participated in its design, performed bioinformatic analyses of the His-to-Asp proteins, and contributed to the development of the manuscript. All authors read and approved the final manuscript.
